# Herbaceous plant diversity mediates saline-alkaline soil improvement in the yellow river delta

**DOI:** 10.3389/fmicb.2025.1734250

**Published:** 2025-12-16

**Authors:** Xiao Wang, Zhaohua Lu, Jingyao Yan, Ge Wang, Xiaohui Chen, Jiangbao Xia

**Affiliations:** 1 School of Chemical and Environmental Engineering, China University of Mining and Technology-Beijing, Beijing, China; 2 Shandong Key Laboratory of Eco–Environmental Science for the Yellow River Delta, Shandong University of Aeronautics, Binzhou, China; 3 Shandong Wudi Gold Turn Land Development and Construction Co., Ltd., Binzhou, China

**Keywords:** salt ions, soil improvement, soil nutrients, plant diversity, Yellow River Delta

## Abstract

**Objectives:**

Soil salinization is a primary constraint on plant colonization and ecosystem stability in the Yellow River Delta. However, there is currently a significant lack of research concerning the improvement of saline-alkaline soil quality in the Yellow River Delta, particularly regarding the enhancement of plant communities in saline-alkaline soil. Therefore, undertaking saline-alkaline soil remediation is particularly crucial for enhancing the ecological adaptability of plant communities.

**Methods:**

Shandong Yellow River Island National Wetland Park, a representative habitat within the Yellow River Delta, were selected as the study area. Moreover, a comprehensive survey of herbaceous plant communities was performed, and the community types, α and β diversity, soil physicochemical properties indicators were determined and analyzed. The effects of different herbaceous plant communities on soil physicochemical properties were investigated. Principal component analysis (PCA) and fuzzy membership functions were used to comprehensively evaluate the improvement effect of different herbaceous plant communities on saline–alkaline soil quality. The study critically evaluated the efficacy of different herbaceous plant communities in improving saline–alkaline soils within the study area.

**Results:**

(1) The study area contained four main formation groups and ten formations. Our findings revealed pronounced functional disparities among the communities: the *Artemisia scoparia* and *Aeluropus sinensis* communities demonstrated high diversity and stability, whereas the *Suaeda salsa* community exhibited low diversity and poor viability. (2) The herbaceous plant community significantly affected the physicochemical properties of the 0–20 cm soil layer. Although soil bulk density (BD) and pH remained unaffected, communities with higher diversity, particularly *A. scoparia* and *S. salsa*, most effectively reduced soil salinity by regulating ions (Na^+^, Cl^–^, SO_4_^2–^). Furthermore, specific communities differentially increased soil nutrientsa*S. salsa*, *A. scoparia*, and *Phragmites australis* communities were important for increasing soil total nitrogen (TN), available phosphorus (AP), and soil organic matter (SOM), respectively. However the proliferation of plant communities exerts a pronounced inhibitory effect on soil available potassium (AK). (3) Principal component analysis (PCA) and composite factor scores ultimately revealed that the *S. salsa*, *A. scoparia*, and *P. australis* communities were the most effective communities for comprehensive soil quality improvement on Yellow River Island.

**Conclusion:**

In summary, it is evident that herbaceous plant communities with high diversity yield the most effective soil salinity reduction and improvement outcomes on Yellow River Island. Coastal saline-alkaline soils exhibit elevated levels of readily available potassium due to external inputs; consequently, the capacity of herbaceous plant communities to reduce AK serves as a crucial criterion for evaluating their soil amelioration efficacy. Furthermore, given the spatial heterogeneity of soil salinity on the Yellow River Island, the configuration of soil-improving plant communities must balance ecological and cost-effectiveness considerations.

## 1 Introduction

The Yellow River Delta, which is located in the coastal area of eastern China, is a region where land and sea intertwine. It was formed through interactions between the Yellow River and the Bohai Sea. Under the effects of Bohai Sea tides and seawater intrusion, the soils in this area exhibit significant salinization characteristics (Kuang et al., 2022). The saline–alkaline land area in the Yellow River Delta has reached 4,657 km^2^, which accounts for 85.45% of the total land area (Pan, 2023). Coastal saline–alkaline soil has the characteristics of soluble salt (SS) ions and a high groundwater table, which can lead to waterlogging and reduce effective soil porosity, thereby impairing water holding capacity, promoting soil compaction, influencing the decomposition efficiency and enzyme activity of soil microbes, and reducing soil fertility (Mansouri et al., 2021) High concentrations of soil salt ions are highly toxic to plants, resulting in severe damage to their physiological structures and inhibition of their antioxidant functions, These characteristics are thereby impacted in plant communities significantly decreasing plant diversity (Salwan et al., 2019; Neupane et al., 2024). Consequently, soil salinization has become a major limiting factor for plant colonization and regional vegetation distribution in the coastal saline–alkaline soils of the Yellow River Delta.

The restoration of soil quality in saline–alkaline land is a critical for both ecological stability and human wellbeing. Healthy soil possesses high fertility, providing plants with an optimal water-nutrient-air-heat environment, and serves as the essential material foundation for plant growth (Zeng et al., 2025). Because healthy soil is a fundamental determinant of ecosystem functionality, It is essential to promote the ecological restoration of fragile habitats and improve the living environments of animals, plants, and humans (Santos et al., 2021). The colonization of dominant plant communities plays a vital role in improving soil quality. In the context of the severely degraded coastal habitats of the Yellow River Delta, soil improvement not only has ecological significance but also directly impacts regional biodiversity conservation and sustainable land management practices. Halophytes, such as *Suaeda salsa*, *Phragmites australis*, and *Tamarix chinensis*, are the dominant species in the Yellow River Delta. These plants can withstand tidal erosion, promote soil and water conservation, enhance habitat climate characteristics, provide stable sediment, accelerate soil water and nutrient cycling, and facilitate the exchange and transport of soil SS ions. As a result, they significantly reduce soil salinity, increase soil fertility, and critically contribute to maintaining ecosystem stability (Mitran et al., 2021; Islam et al., 2022; Yu et al., 2023). Therefore, selecting and rationally allocating appropriate plant species are effective measures for improving the soil quality of coastal saline–alkaline land in the Yellow River Delta. The elucidation of the soil improvement effect of different herbaceous plant communities is important for understanding the ecological adaptability of plants.

Previous studies on the relationships between plant communities and soil in saline–alkaline land have focused primarily on the response patterns of plant physiological structures and functions to soil salinity stress (Mori et al., 2011; Paul et al., 2025), the ecological adaptation characteristics of plants to salinity stress (Shahid et al., 2020; Rozentsvet et al., 2021), the response patterns of soil microbes and enzymes as plants resist salinity stress (Roy et al., 2021; Mahmoud et al., 2024), and the effects of shrub communities such as *T. chinensis* on the soil quality of saline–alkaline land (Chen et al., 2022; Ambrosino et al., 2023). While woody species have traditionally received more research attention, emerging evidence highlights the pivotal role of herbaceous communities in coastal saline–alkaline environments. Herbaceous plants possess formidable reproductive capacity and rapid growth rates, thereby dominating soil seed banks (Mmusi et al., 2021). Concurrently, these plants typically exhibit shorter life cycles (generally less than 3 years), enabling their plant debris to rapidly transform into soil organic matter and enhance soil fertility (Luo et al., 2025). These factors collectively position herbaceous plants as the species of choice for ameliorating saline-alkaline soil quality. Herbaceous plants are adept at breaking the positive feedback loops between soil salinity and vegetation degradation that characterize these fragile ecosystems. However, research on the mechanisms by which different herbaceous plant communities improve saline–alkaline soil quality remains insufficient. In particular, studies examining how various herbaceous plant communities ameliorate coastal saline–alkaline soils are lacking. To address this gap, we selected Yellow River Island, a typical national wetland park in the Yellow River Delta, as our study area and focused on naturally growing herbaceous plant communities. Furthermore, the α and β diversity indices of different herbaceous plant communities and their soil physicochemical characteristics were analyzed, and the effects of different herbaceous plant communities on soil quality improvement were evaluated. The innovative aspects of this research are as follows: (1) quantitative assessment of the effect of dominant herbaceous communities on coastal saline-alkaline soil quality; (2) preliminary investigation into the mechanisms underlying soil salinity reduction and nutrient improvement; (3) identification of optimal plant communities for different salinity conditions; and (4) development of optimized vegetation configuration patterns for saline-alkaline soil remediation. On the basis of the above approach, this research can serve as an important reference for plant selection and allocation in coastal saline–alkaline land in the Yellow River Delta.

## 2 Materials and methods

### 2.1 Study area overview

The study area was located on Yellow River Island in northeastern Wudi County, Binzhou City, Shandong Province (37°54′58.6″ N–38°0′14.908″ N, 118°1′0.204″ E–118°4′2″ E), which is part of the Yellow River Island National Wetland Park. This region is a typical representative area of coastal saline–alkaline land in the Yellow River Delta, with an area of 25.96 km^2^. The study area has a warm temperate monsoon climate, with an average annual temperature of 13.1 °C, an average annual precipitation of 493 mm, an annual average humidity of 65%, an evaporation–precipitation (E/P) ratio of 3.97:1, and an annual average wind speed of 3.0 m/s. The terrain of the study area generally tends to be high in the south and low in the north. Under the effects of tidal impact and river sedimentation, the terrain has a banded distribution with an average elevation of 0.2–3.4 m. The average depth to the groundwater table is 1.3 m. The soil types are coastal salinized Fluvo–aquic soil and coastal alluvial–salt soil, with coastal salinized Fluvo–aquic soil being dominant. The average salinity is 7.49 g/L. The vegetation in the study area is characterized by the interlaced growth of nonhalophyte and halophyte communities. The main plants are *S. salsa*, *P. australis*, *Aeluropus sinensis*, *Artemisia scoparia*, *Setaria viridis*, *Taraxacum mongolicum* (dandelion), *Glycine soja* (wild soybean), and *Sonchus wightianus*, among others. The animal species include mainly protected species, such as common cranes and whooper swans.

### 2.2 Experimental design

**FIGURE 1:**
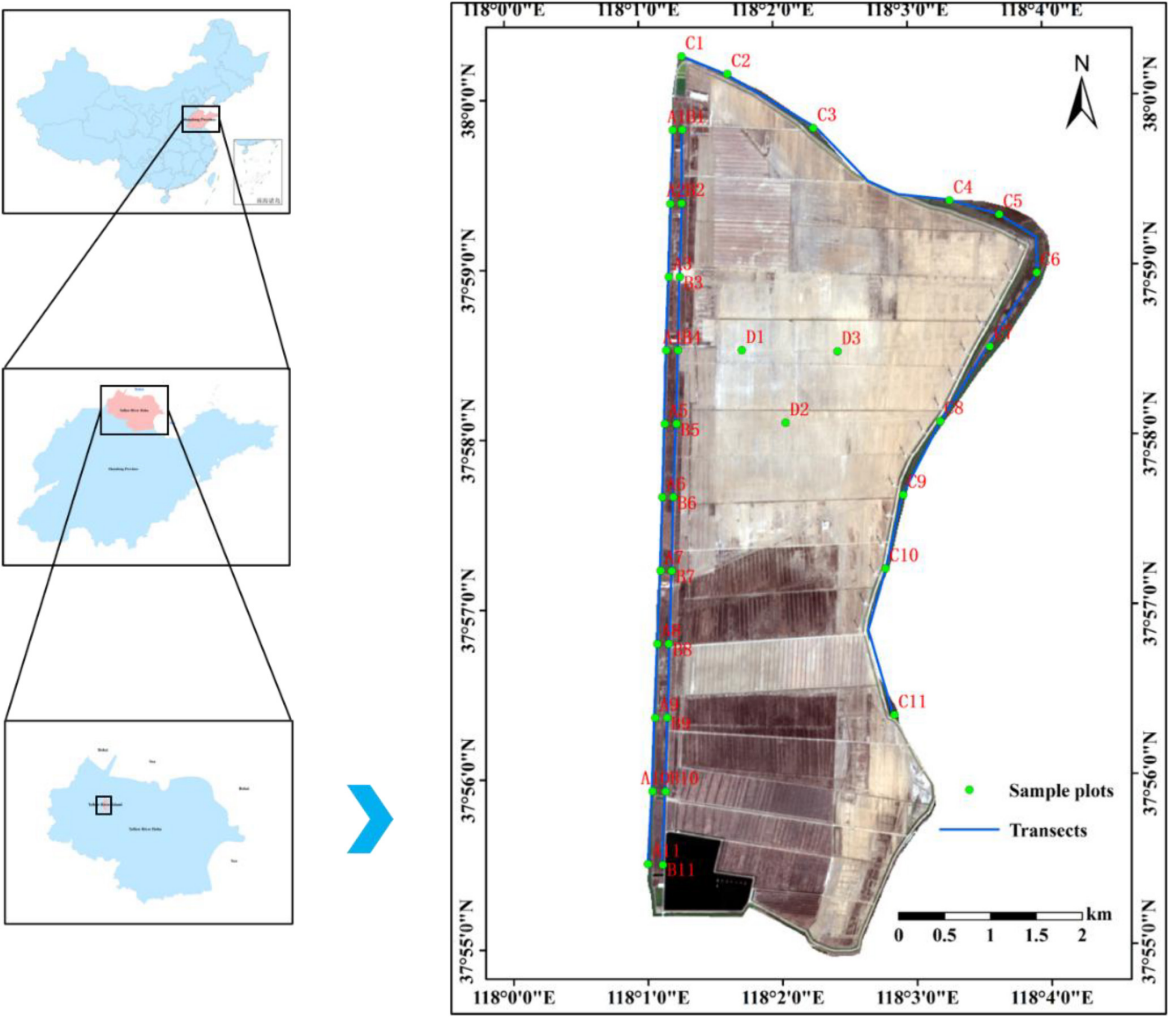
Distribution map of sample plots and transect locations on Yellow River Island. The green dots in the map indicate the locations of the 36 sample plots, the red letters and numbers denote the plot numbers, and the blue lines represent the three transects.

A field plant community survey was conducted on Yellow River Island from July to August 2023. A total of 3 transects were established according to the distances to the coastal zone and the riparian zone, 11 sample plots were established at equal intervals for each transect, 3 samples were collected for each sample plot, and the spacing between the sample plots was 800 m (Figure 1). Three plots (D1, D2, and D3) were established adjacent to the bare soil area to serve as the control group (CK). A total of 36 sample plots were established, and each sample plot was 30 m × 30 m in size. Five 1 m × 1 m herbaceous quadrats were established in each sample plot using the five–point method, for a total of 165 quadrats. In each plot, the “S” sampling method was used to establish five soil sampling points, and soil samples from the two soil layers, 0–20 cm and 20–40 cm, were collected. Soil was collected using a ring knife (100 m^3^) and an aluminum box (55 mm × 35 mm) to determine soil physical indicators. Moreover, 500 g soil samples were collected in ziplock bags, and were subsequently designated for the determination of salinity and nutrient indicators.

### 2.3 Determination methods

In the plant community survey, the names of herbaceous plants and the quantity indicators of the communities in the quadrats were recorded, and the importance value (IV) and α diversity index of the plant communities were calculated (Kumar et al., 2010). The soil water content (SWC) and bulk density (BD) were determined by the oven drying (105°C, 8 h) and ring knife methods, respectively (Bao, 2000). Soil pH was measured using the electrode method, the soil SS content was determined through the gravimetric method (Bao, 2000), the main cation contents of soil K^+^, Ca^2+^, Na^+^, and Mg^2+^ were determined via atomic absorption spectrometry (Bao, 2000), and silver nitrate, ethylenediaminetetraacetic acid (EDTA) and sulfuric acid standard solution titration methods were used to determine the contents of the main anions in soil, such as Cl^–^, SO_4_^2–^, CO_3_^2–^, and HCO_3_^–^ (Bao, 2000). The soil organic carbon (SOC) content was determined using the potassium dichromate oxidation method (Bao, 2000), the soil total nitrogen (TN) content was determined via the element analyzer method (Lv et al., 2023), the soil available phosphorus (AP) content was determined through the molybdenum–antimony resistance colorimetric method (Bao, 2000), and the soil available potassium (AK) content was determined with flame photometry (Bao, 2000).

### 2.4 Statistical analysis

The plant and soil data were analyzed by one–way analysis of variance (ANOVA) and least significant difference (LSD) multiple comparisons using SPSS 25. The composition of the plant communities was obtained from TWINSPAN analysis using the IV of the plant communities, and PC–ORD software was used to complete the analysis (Grandin, 2006). β diversity calculations were performed using the Bray–Curtis distance–based non–metric multidimensional scaling (NMDS) analysis method, and Canoco 5 software was used to complete the analysis (Legendre and Legendre, 2012). Principal component analysis (PCA) and fuzzy membership functions were used to evaluate the effects of different herbaceous plant communities on soil quality. The detailed calculation process can be expressed as follows.

According to the principal component accounted for by each soil factor, if all the soil factors contained in the i–th principal component had a positive effect (promoting effect) on the soil quality, the degree of membership was calculated by Equation 1:


$$U_{j}^{\left(i\right)}=\frac{X_{j}^{\left(i\right)}-X_{m\,i\,n}^{\left(i\right)}}{X_{m\,a\,x}^{\left(i\right)}-X_{m\,i\,n}^{\left(i\right)}}$$ (1)

If all the soil factors contained in the i–th principal component had a negative effect (inhibition effect) on the soil quality, the degree of membership could be expressed (Equation 2):


$$U_{j}^{\left(i\right)}=1-\frac{X_{j}^{\left(i\right)}-X_{m\,i\,n}^{\left(i\right)}}{X_{m\,a\,x}^{\left(i\right)}-X_{m\,i\,n}^{\left(i\right)}}$$ (2)

The weights of the principal components and the comprehensive score of the sample plots are calculated according to Equations 3, 4.


$$W_{i}=\frac{m_{i}}{\sum_{i=1}^{n}m_{i}}$$ (3)

$$D_{j}=\sum_{i=1}^{n}W_{i}\,U_{j}^{\left(i\right)}$$ (4)

where $U_{j}^{\left(i\right)}$ is the membership degree of the j–th plot on the i–th principal component; i is the effective principal component number obtained from PCA, i = 1, 2, 3,…n; n is the maximum number of principal components; j is the sample plot number, j = 1, 2, 3…k, where k is the total number of sample plots; $X_{j}^{\left(i\right)}$ is the factor score of the j–th sample plot on the i–th principal component; $X_{m\,i\,n}^{\left(i\right)}$ is the minimum factor score on the i–th principal component; $X_{m\,a\,x}^{\left(i\right)}$ is the maximum factor score on the i–th principal component; *W_i_* is the weight occupied by the i–th principal component; *m_i_* is the eigenvalue of the cumulative variance of rotated component loadings of the i–th principal component; and *D_j_* is the composite score of the j–th plot. The composite score of the community was computed as the mean of the composite scores of the plots belonging to one community type.

## 3 Results

### 3.1 Types of plant communities

**FIGURE 2:**
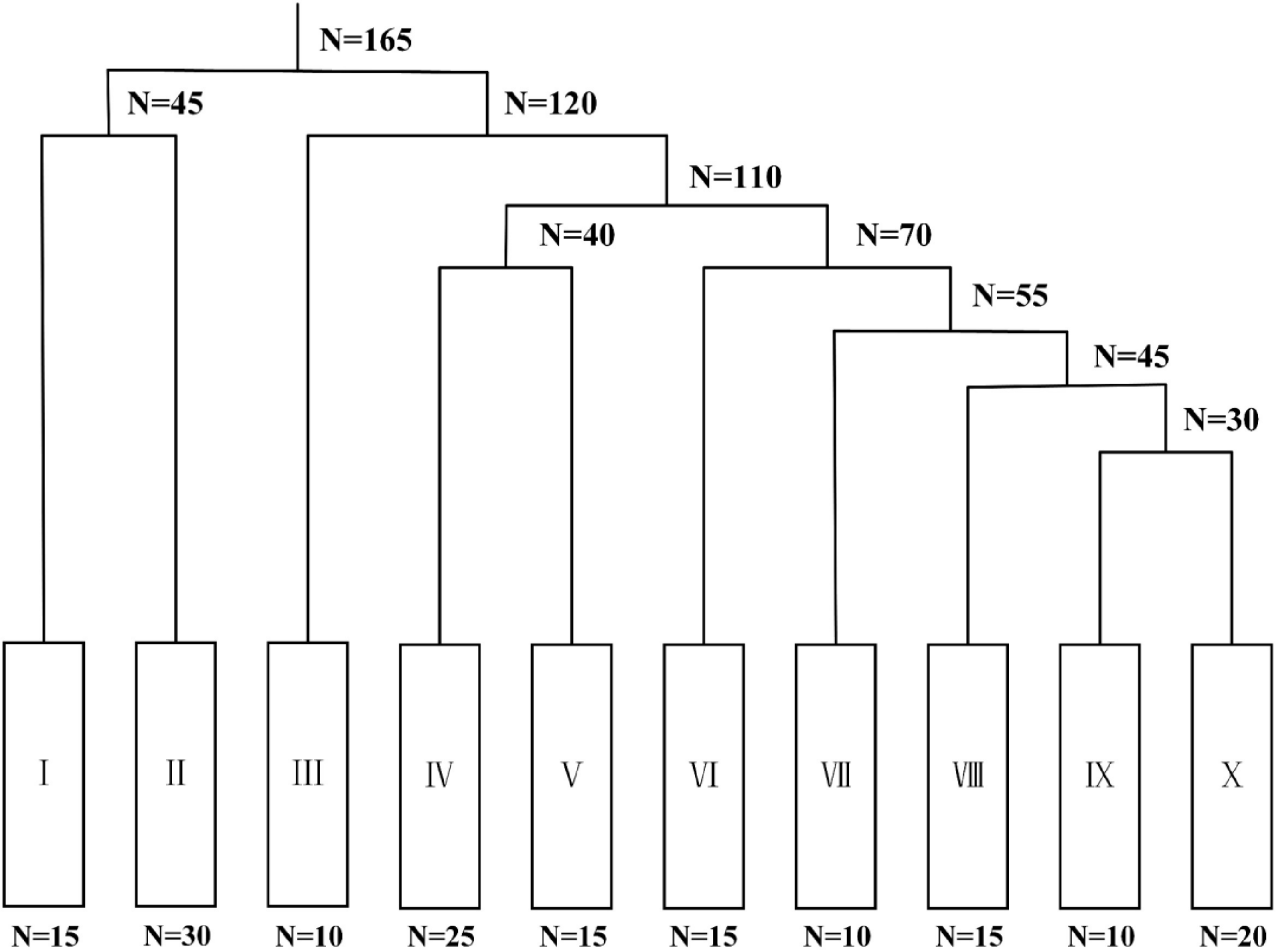
TWINSPAN classification of major plant community types in the study area, where I is the *Suaeda salsa* formation; II is the *Suaeda salsa–Spartina alterniflora* formation; III is the *Aeluropus sinensis formation*; IV is the *Artemisia scoparia* formation; V is the *Artemisia scoparia–Imperata cylindrica* formation; VI is the *Phragmites australis* formation; VII is the *Phragmites australis–Suaeda salsa* formation; VIII is the *Phragmites australis–Aeluropus sinensis* formation; IX is the *Phragmites australis–Imperata cylindrica* formation; and X is the *Phragmites australis–Cynodon dactylon* formation. N denotes the number of sample plots in each distinct group at each level following partitioning, the number following the equal sign denotes the specific quantity of sample plots.

The plant community survey identified a total of 24 herbaceous plant species, which were classified into 20 genera and 9 families. The classification results of the main plant communities using the TWINSPAN method are shown in Figure 2. On the basis of the classification criteria, five classifications (0–2, 2–5, 5–10, 10–50, and > 50) were established, and seven levels were used for the analysis. The herbaceous vegetation types in the study area were categorized into four formation groups and 10 formations. Specifically, the *S. salsa* formation group included two formations: *S. salsa* (I) and *S. salsa–S. alterniflora* (II). The *A. sinensis* formation group contained one formation: *A. sinensis* (III). The *A. scoparia* formation group consisted of two formations: *A. scoparia* (IV) and *A. scoparia–I. cylindrica* (V). The *P. australis* formation group included five formations: *P. australis* (VI), *P. australis–S. salsa* (VII), *P. australis–A. sinensis* (VIII), *P. australis–I. cylindrica* (IX), and *P. australis–C. dactylon* (X).

### 3.2 Plant diversity

#### 3.2.1 Plant α diversity

The herbaceous plant communities in the 33 sampling plots were classified into four community types on the basis of the formation group types. The diversity indices for these communities are presented in Table 1. On the basis of the Simpson index, the communities were ranked as follows: *S. salsa* (SSA) < *P. australis* (PAU) < *A. scoparia* (ASC) < *A. sinensis* (ASI). The Simpson index values ranged from 0.45 to 0.71. Both ASI and ASC exhibited significantly higher Simpson index values by 57.78 and 37.78%, respectively, compared with SSA (*p* < 0.05). No significant differences were detected among the other communities (*p* > 0.05). The variation patterns of the Shannon–Wiener index and the Simpson index were consistent. On the basis of the Pielou evenness index, the communities were ranked as follows: PAU < ASC < ASI < SSA, with values ranging from 0.55 to 0.82. The Pielou evenness index values for PAU and ASC (32.93 and 25.61%, respectively) were significantly lower than those for SSA (*p* < 0.05). The differences among the other communities were not significant (*p* > 0.05). On the basis of the Margalef richness index, the communities were ordered as follows: SSA < PAU < ASI < ASC, with values ranging from 0.25 to 1.45. The Margalef richness index values for ASC, ASI, and PAU were significantly higher by 4.80, 4.48, and 3.52 times, respectively, than those for SSA (p < 0.05). The analysis revealed that the *A. scoparia* and *A. sinensis* communities exhibited high species richness and low dominance, indicating high community stability. In contrast, the *S. salsa* community had the lowest species richness but high species evenness and strong stability. The *P. australis* community had low species richness and the lowest species evenness, coupled with high dominance and poor stability. Additionally, the PAU community was easily replaced by other communities through competition.

**TABLE 1:** Diversity indices of herbaceous plant communities.

Community name	Simple plot number	Simpson index	Shannon–Wiener index	Pielou evenness index	Margalef richness index
*S. salsa* (SSA)	C1, C2, C3, C4, C5, C6, C7, C8, C9, C10	0.45 ± 0.03^a^	0.67 ± 0.03^a^	0.82 ± 0.05^b^	0.25 ± 0.03^a^
*A. sinensis* (ASI)	A3, A4, B4, A5, B5, B7	0.71 ± 0.03^b^	1.49 ± 0.10^b^	0.69 ± 0.04^ab^	1.37 ± 0.13^b^
*A. scoparia* (ASC)	B2, A7, A8, B8, A9, B9, A10, B10	0.62 ± 0.05^b^	1.35 ± 0.14^b^	0.61 ± 0.05^a^	1.45 ± 0.12^b^
*P. australis* (PAU)	A1, B1, A2, B3, A6, B6, A11, B11, C11	0.55 ± 0.09^ab^	1.12 ± 0.19^ab^	0.55 ± 0.08^a^	1.13 ± 0.21^b^

1. The letters in parentheses indicate community codes. 2. Simple Plot Number derived from Figure 1. 3. The superscript letters in the same column indicate the significance of the data on the basis of ANOVA, with the same letters indicating that the difference is not significant (*p* > 0.05) and different letters indicating that the difference is significant (*p* < 0.05).

#### 3.2.2 Plant β diversity

**FIGURE 3:**
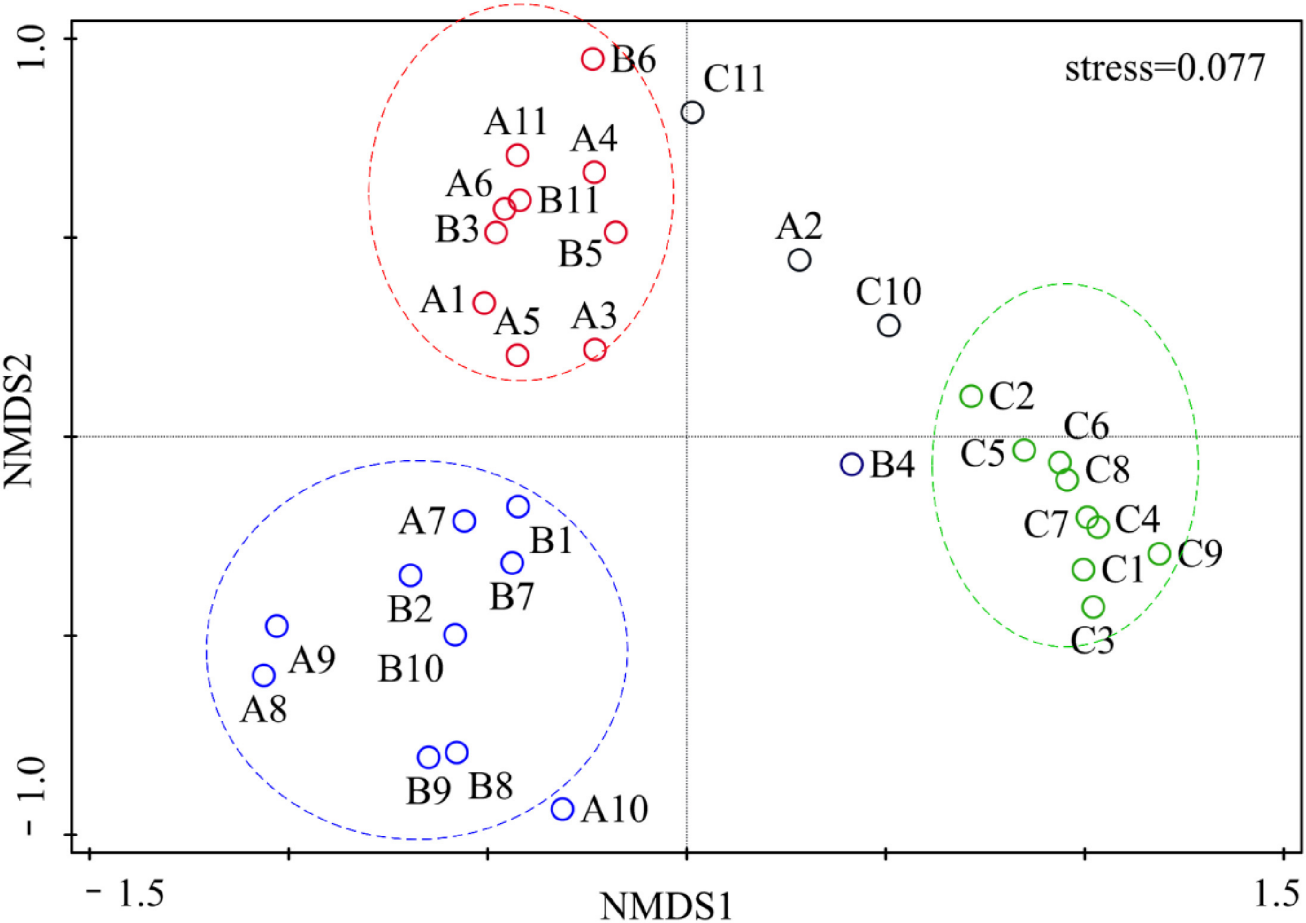
NMDS ranking results of the β diversity of the herbaceous plant communities. The horizontal and vertical axes represent the first two ranking axes of the NMDS analysis. Solid small circles of varying colors denote study plots, with alphanumeric combinations representing plot numbers. The distance between two solid small circles indicates the Bray-Curtis distance between plots, reflecting their degree of dissimilarity. Large circles with dashed borders of differing colors represent the clustering groups of plots within the NMDS results.

β diversity reflects the similarity in the distribution of plant communities among different habitats. Bray–Curtis dissimilarity was used to evaluate the similarity between different plant communities. As shown in Table 2, the Bray–Curtis dissimilarity between the SSA and ASC was the highest at 0.9746, indicating the lowest similarity between these communities. The Bray–Curtis dissimilarity between the SSA and both the ASI and PAU was greater than 0.9, indicating relatively low similarity between the SSA and the other three communities. The dissimilarity between the PAU and ASI was the lowest at 0.5807, indicating the highest similarity between these communities. Furthermore, the Bray–Curtis dissimilarities between ASC and PAU, and between ASC and ASI were 0.6731 and 0.7601, respectively, indicating relatively high similarity between ASC and the other two communities. The NMDS ranking diagram of the various sampling plots in the study area based on the Bray–Curtis dissimilarity was shown in Figure 3. The stress value of the NMDS analysis was 0.077, which indicated that the high reliability of the results. As shown in Figure 3 and Table 2, the herbaceous plant communities were divided into three distinct groups. One group was composed mainly of *P. australis* and *A. sinensis* communities, another group was composed mainly of *A. scoparia* communities, and the other group was composed mainly of *S. salsa* communities. The result of analysis revealed that the vegetation distributions of the *S. salsa* community significantly differed from those of the other three communities. In contrast, the differences in herbaceous distribution among the *A. scoparia*, *P. australis* and *A. sinensis* communities were relatively low, with the greatest similarity observed between the *P. australis* and *A. sinensis* communities.

**TABLE 2:** Matrix of the Bray–Curtis dissimilarities among different plant communities.

Community name	SSA	ASI	ASC	PAU
SSA	0	0.9316	0.9746	0.9202
ASI	0.9316	0	0.7601	0.5807
ASC	0.9746	0.7601	0	0.6731
PAU	0.9202	0.5807	0.6731	0

### 3.3 Soil water and physical indicators

**FIGURE 4:**
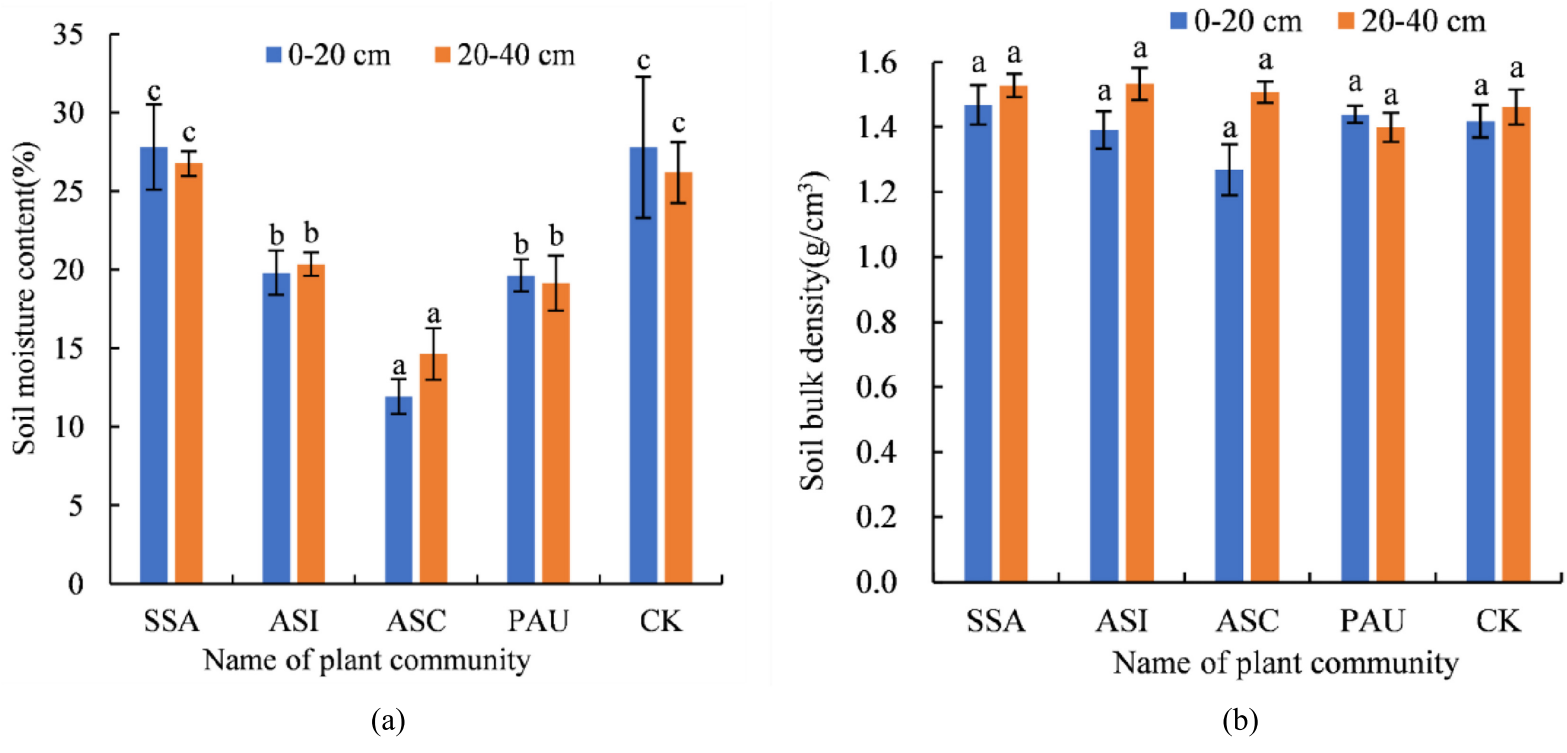
SWC (a) and BD (b) of different herbaceous plant communities in the 0–20 and 20–40 cm soil layers. In this figure, the same letter indicates a nonsignificant difference (*p* > 0.05), and a different letter indicates a significant difference (*p* < 0.05). SSA denotes the *S. Salsa* community, ASI denotes the *A. sinensis* community, ASC denotes the *A. scoparia* community, and PAU denotes the *P. australis* community.

The SWC and BD values of the different herbaceous plant communities are shown in Figure 4. The changes in the SWC of the 0–20 cm soil layer followed the order of ASC < PAU < ASI < CK < SSA, with a range of 11.93–27.81%. The SWC values for ASI, PAU, and ASC were significantly lower than those for CK (*p* < 0.05), decreasing by 28.75, 29.33, and 57.07%, respectively. In contrast, the SSA was not significantly different from the CK (*p* > 0.05). The variation in the SWC in the 20–40 cm soil layer was consistent with that in the 0–20 cm soil layer, with values ranging from 14.65 to 26.76%. Similar to the 0–20 cm layer, the SWC values for ASI, PAU, and ASC were all significantly lower than those for CK (*p* < 0.05), representing reductions of 22.34, 26.88, and 44.06%, respectively. No significant difference in SWC was observed between the two soil layers (*p* > 0.05).

The variation pattern of BD in the 0–20 cm soil layer followed the order of ASC < ASI < CK < PAU < SSA, with a range of 1.27–1.47 g/cm^3^. The BD values of the four plant communities were not significantly different from those of the CK (*p* > 0.05). However, the BD values of SSA and PAU were significantly greater than those of ASC (*p* < 0.05), demonstrating increases of 15.75 and 13.39%, respectively. In the 20–40 cm soil layer, BD values followed the order of PAU < CK < ASC < SSA < ASI, with a range of 1.40–1.53 g/cm^3^. The BD values of the four plant communities significantly differed from those of the CK (*p* > 0.05), but those of the SSA and ASI were significantly greater than those of the PAU (*p* < 0.05), demonstrating increases of 9.10 and 9.48%, respectively. With respect to ASC, the BD value in the 0–20 cm soil layer was significantly lower than that in the 20–40 cm soil layer (*p* < 0.05). Compared with that in the 20–40 cm soil layer, the BD value in the 0–20 cm soil layer decreased by 15.89%, and no significant differences were observed between the other plant communities and the CK (*p* > 0.05).

### 3.4 Soil salinity and alkalinity indicators

**FIGURE 5:**
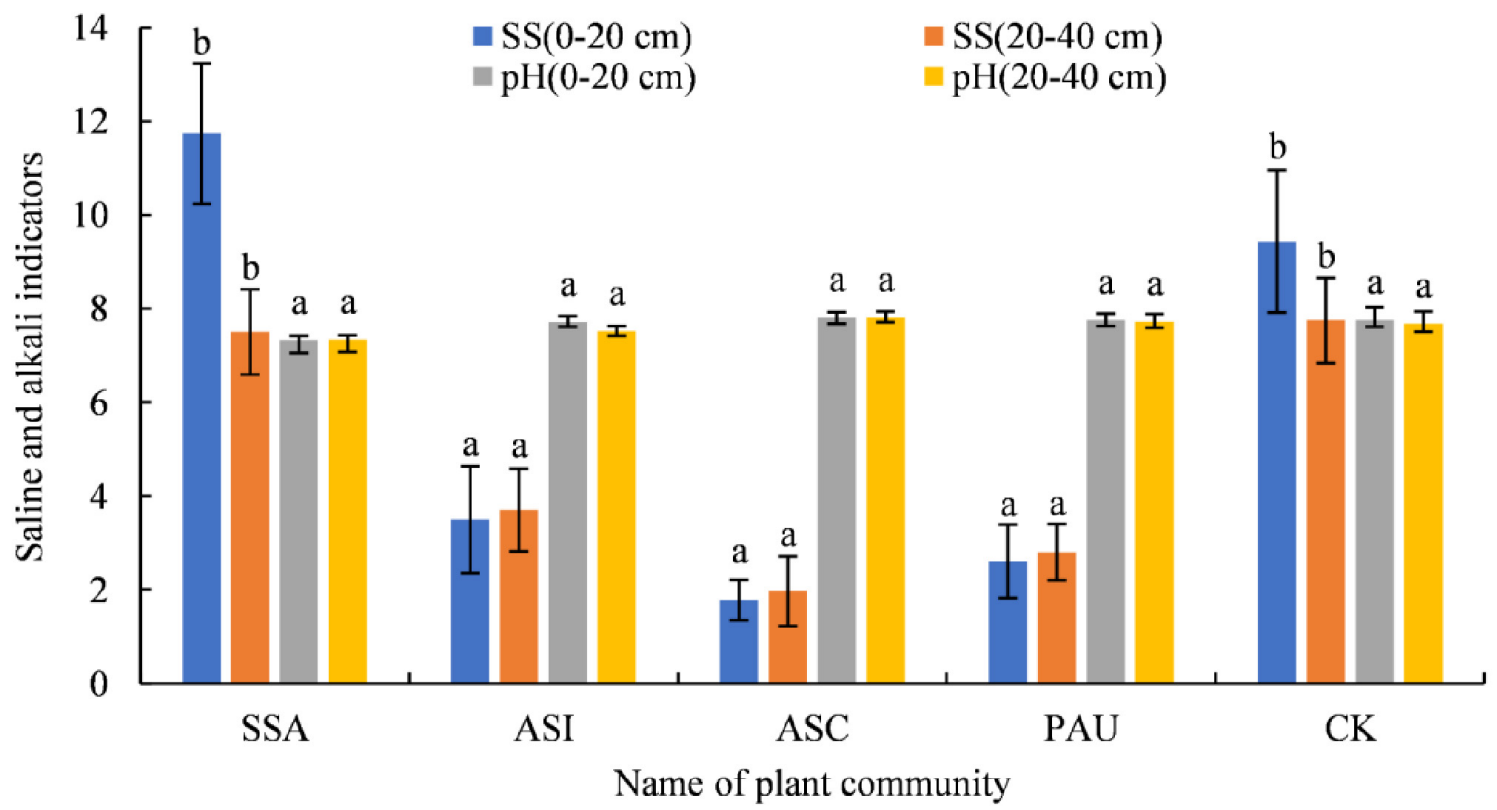
Soil pH and SS content of different herbaceous plant communities. In this figure, the same letter indicates a nonsignificant difference (*p* > 0.05), and a different letter indicates a significant difference (*p* < 0.05). SSA denotes the *S. Salsa* community, ASI denotes the *A. sinensis* community, ASC denotes the *A. scoparia* community, and PAU denotes the *P. australis* community.

The soil pH and SS content values of the different herbaceous plant communities are shown in Figure 5. The soil pH ranged from 7.32 to 7.82, and no significant differences were observed between the different plant communities and soil layers (*p* > 0.05). The SS content in the 0–20 cm soil layer followed the order of ASC < PAU < ASI < CK < SSA, with values ranging from 1.78 to 11.74 g/kg. The SS values in ASC, PAU, and ASI were significantly lower than that in CK (*p* < 0.05), with reductions of 81.14, 72.35, and 63.03%, respectively. In the 20–40 cm soil layer, the SS values followed the order of ASC < PAU < ASI < SSA < CK, with a range of 1.97–7.51 g/kg. The SS values in ASC, PAU, and ASI were significantly lower than that in CK (*p* < 0.05), with reductions of 74.58, 63.87, and 52.13%, respectively. With respect to the SSA community, the SS value in the 0–20 cm soil layer was significantly greater than that in the 20–40 cm layer (*p* < 0.05), demonstrating an increase of 56.32%. No significant differences were detected between the other plant communities and the CK (*p* > 0.05).

### 3.5 Soil salt ion contents

#### 3.5.1 Soil cation contents

**FIGURE 6:**
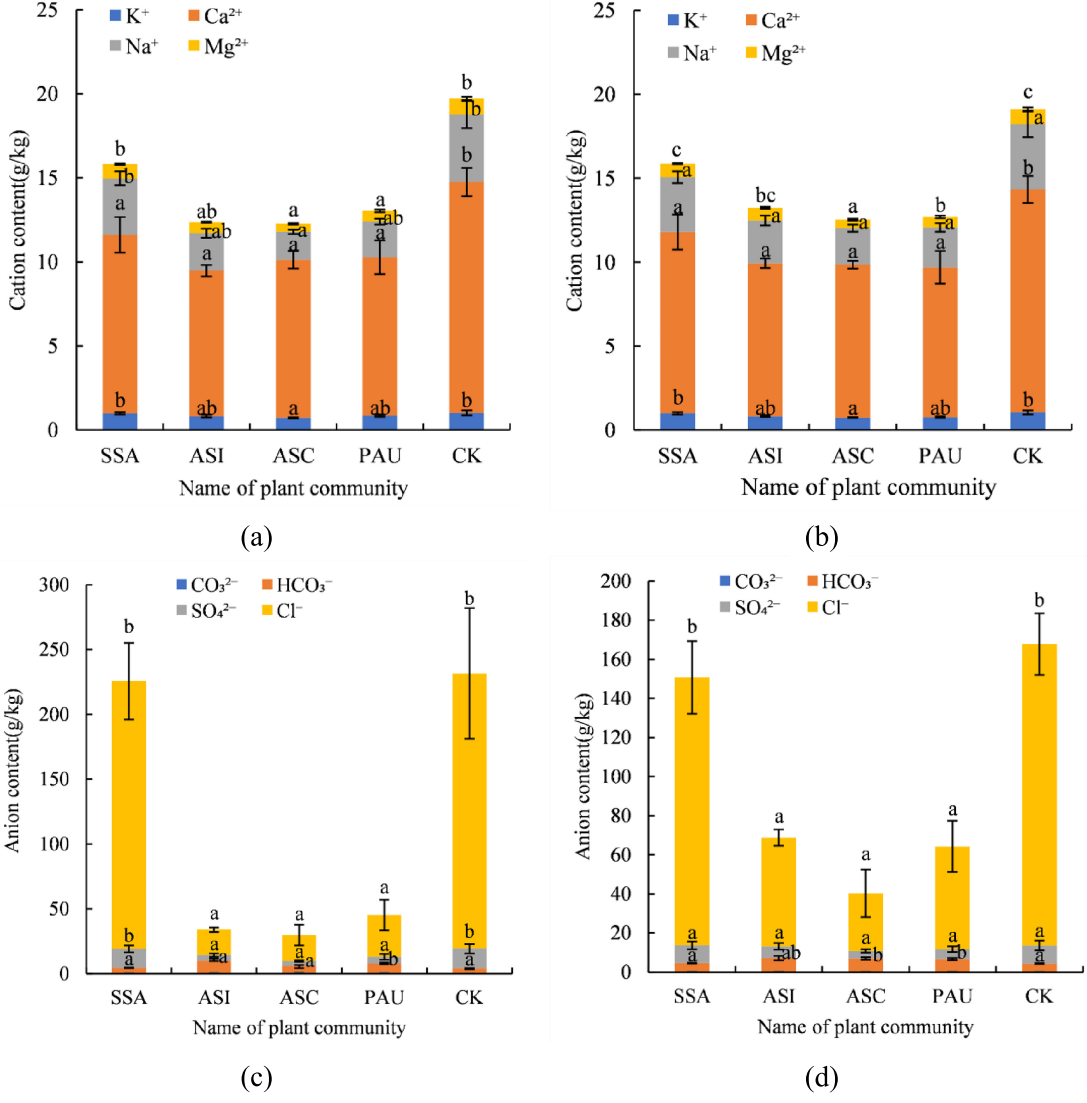
Soil salt ion contents of different herbaceous plant communities: (a) soil cation contents in the 0–20 cm soil layer; (b) soil cation contents in the 20–40 cm soil layer; (c) soil anion contents in the 0–20 cm soil layer; and (d) soil anion contents in the 20–40 cm soil layer. In this figure, the same letter indicates a nonsignificant difference (*p* > 0.05), and a different letter indicates a significant difference (*p* < 0.05). SSA denotes the *S. Salsa* community, ASI denotes the *A. sinensis* community, ASC denotes the *A. scoparia* community, and PAU denotes the *P. australis* community.

The soil cation contents of the different herbaceous plant communities are shown in Figure 6. The main cations associated with soil salinity were Na^+^ and Ca^2+^. In the 0–20 cm soil layer, the Na^+^ content followed the order of ASC < ASI < PAU < SSA < CK, and the range was 1.67–4.02 g/kg. The content in the ASC was significantly lower than that in the CK (*p* < 0.05), demonstrating a 58.46% reduction. The Na^+^ content in the 20–40 cm soil layer followed the order of ASC < PAU < ASI < SSA < CK, and the range was 2.18–3.89 g/kg. No significant differences were observed in the soil Na^+^ contents between any of the plant communities (*p* > 0.05). The Ca^2+^ content in the 0–20 cm soil layer followed the order of ASI < ASC < PAU < SSA < CK, and the range was 8.66–13.74 g/kg. Compared with those in the CK community, the contents in SSA, PAU, ASC, and ASI communities decreased by 22.71, 31.37, 31.51, and 36.97%, respectively. The Ca^2+^ content in the 20–40 cm soil layer followed the order of PAU < ASI < ASC < SSA < CK, and the range was 8.66–13.29 g/kg. The contents in all the plant communities were significantly lower than those in the CK community (*p* < 0.05). Specifically, the Ca^2+^ contents in SSA, ASC, ASI, and PAU decreased by 18.74, 31.41, 31.44, and 32.88%, respectively. The contents of other cations, such as K^+^ and Mg^2+^, were also low.

#### 3.5.2 Soil anion contents

The soil anion contents of the different herbaceous plant communities are shown in Figure 6. The main anions associated with soil salinity were SO_4_^2–^ and Cl^–^. In the 0–20 cm soil layer, the SO_4_^2–^ contents followed the order of ASC < ASI < PAU < SSA < CK, and the range was 3.65–15.13 g/kg. The contents in the ASC, ASI, and PAU were significantly lower than those in the CK (*p* < 0.05), with reductions of 75.88, 71.51, and 66.42%, respectively. The SO_4_^2–^ content in the 20–40 cm soil layer followed the order of ASC < PAU < ASI < SSA < CK, and the range was 3.79–9.20 g/kg. Only the content in the ASC was significantly lower than that the CK (*p* < 0.05), showing a 58.80% reduction. Moreover, no significant differences were detected between the other communities and the CK (*p* > 0.05). There were no significant differences in the soil SO_4_^2–^ contents between the different soil layers (*p* > 0.05). The Cl^–^ content in the 0–20 cm soil layer followed the order of ASI < ASC < PAU < SSA < CK, and the range was 9.60–212.26 g/kg. The contents of ASI, ASC, and PAU were significantly lower than those in the CK (*p* < 0.05), with decreases of 90.77, 90.53, and 66.42%, respectively. In the 20–40 cm soil layer, the soil Cl^–^ content followed the order of ASC < PAU < ASI < SSA < CK, and the range was 29.57–154.15 g/kg. The contents in the ASC, PAU, and ASI were all significantly lower than those in the CK (*p* < 0.05), with decreases of 80.82, 65.85, and 63.89%, respectively. With respect to the ASI and PAU, the soil Cl^–^ concentration in the 0–20 cm soil layer was significantly lower than that in the 20–40 cm soil layer (*p* < 0.05). Compared with that in the 20–40 cm soil layer, the soil Cl^–^ content in the 0–20 cm soil layer decreased by 64.79 and 38.85%, respectively. No significant differences were detected between the other plant communities and the CK (*p* > 0.05). The contents of other anions, such as CO_3_^2–^ and HCO_3_^–^, were low.

In summary, the overall soil salinization level in the study area was high. The contents of soil salt ions in the 0–20 cm soil layer were greater than those in the 20–40 cm soil layer, and the major soil salt types were sodium chloride (NaCl) and calcium sulfate (CaSO_4_).

### 3.6 Soil available nutrient contents

**FIGURE 7:**
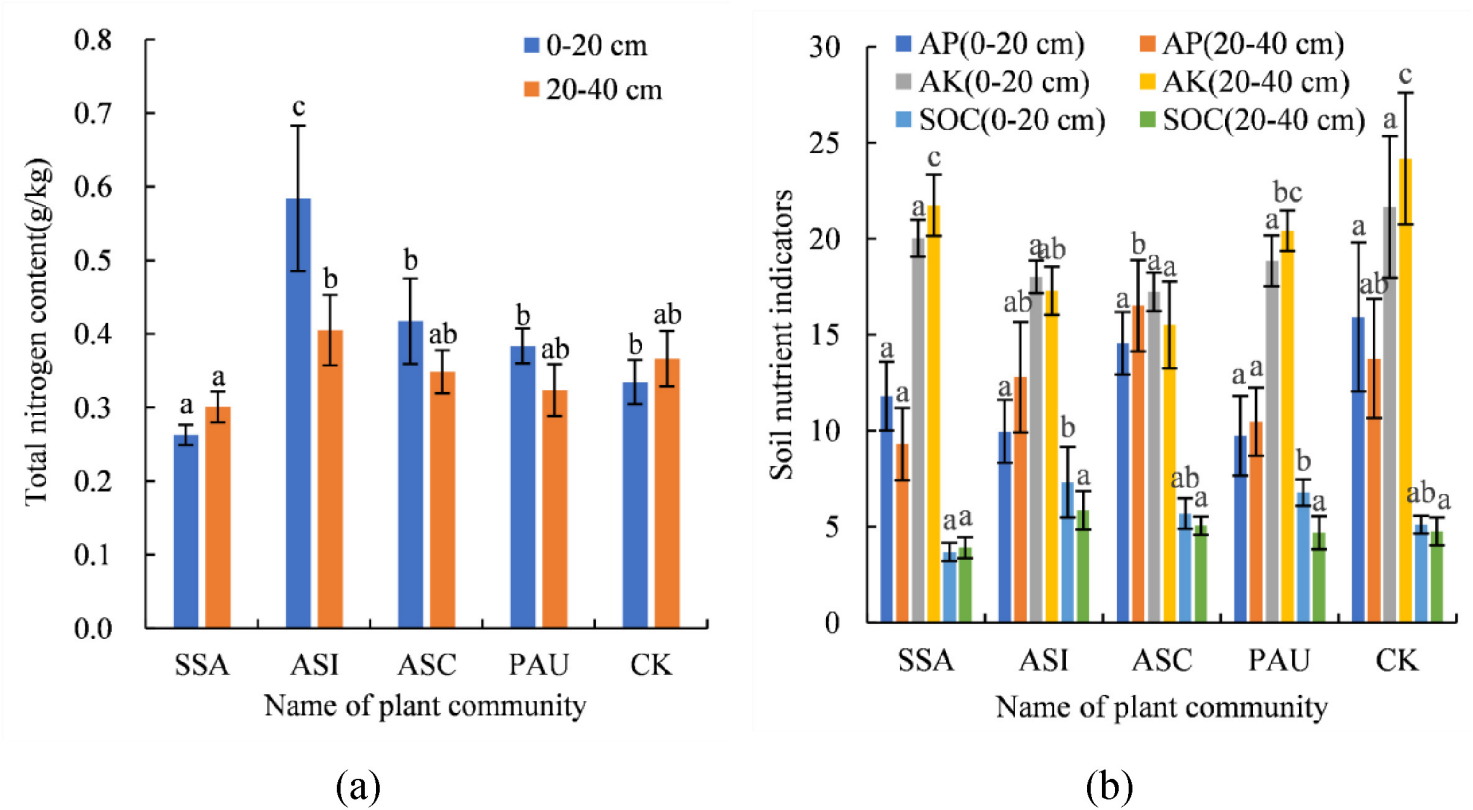
Soil TN content (a) soil AP, available potassium (AK), and SOC contents (b) of different herbaceous plant communities. In this figure, the same letter indicates a nonsignificant difference (*p* > 0.05), and a different letter indicates a significant difference (*p* < 0.05). SSA denotes the *S. Salsa* community, ASI denotes the *A. sinensis* community, ASC denotes the *A. scoparia* community, and PAU denotes the *P. australis* community.

The soil available nutrient contents of the different herbaceous plant communities are shown in Figure 7. In the 0–20 cm soil layer, the soil TN content followed the order of SSA < PAU < ASC < CK < ASI, with a range of 0.26–0.58 g/kg. The TN content in ASI was significantly higher than that in the other plant communities and the CK community (*p* < 0.05). Conversely, the TN content in the SSA was significantly lower than those in the other plant communities and the CK community (*p* < 0.05). Compared with CK, the TN content in ASI increased by 75.76%, while that in SSA decreased by 21.21%. In the 20–40 cm soil layer, the soil TN content followed the order of SSA < CK < PAU < ASC < ASI, with values ranging from 0.30 to 0.41 g/kg. Only in ASI was the TN content significantly higher than that in SSA (*p* < 0.05), demonstrating an increase of 36.67%. The soil TN content in the 0–20 cm soil layer was significantly higher than that in the 20–40 cm soil layer (*p* < 0.05). Compared with that in the 20–40 cm soil layer, the soil TN content in the 0–20 cm soil layer increased by 41.46%. The TN contents in the other plant communities were not significantly different from those in the CK (*p* > 0.05).

The soil AP content in the 0–20 cm soil layer followed the order of PAU < ASI < SSA < ASC < CK, with values ranging from 9.74 to 15.94 mg/kg. In the 20–40 cm soil layer, the soil AP content followed the order of SSA < PAU < ASI < CK, with a range of 9.32–16.52 mg/kg. The AP contents in SSA and PAU were significantly lower than those in ASC (*p* < 0.05), with decreases of 43.58 and 36.56%, respectively. The soil AK content in the 0–20 cm soil layer followed the order of ASC < ASI < PAU < SSA < CK, with values ranging from 17.25 to 21.66 mg/kg. In the 20–40 cm soil layer, the soil AK content followed the order of ASC < ASI < PAU < SSA < CK, with a range of 15.53–24.18 mg/kg. The AK contents in the ASC and ASI treatments were significantly lower than those in the CK treatment (*p* < 0.05), with decreases of 35.77 and 28.49%, respectively. No significant differences were detected between the contents of the other plant communities and those of the CK (*p* > 0.05). The SOC content in the 0–20 cm soil layer followed the order of SSA < CK < ASC < PAU < ASI, with a range of 3.69–7.33 g/kg. The contents in the PAU and ASI were significantly greater than those in the SSA (*p* < 0.05), exhibiting increases of 83.74 and 98.64%, respectively. The SOC content in the 20–40 cm soil layer followed the order of SSA < PAU < CK < ASC < ASI, with values ranging from 3.92 to 5.86 g/kg. With respect to PAU, the SOC content in the 0–20 cm soil layer was significantly higher than that in the 20–40 cm layer, demonstrating an increase of 44.56%. In summary, compared with those in the other communities, the soil nutrient contents in the *A. scoparia* and *A. sinensis* communities were increased, and the nutrient contents in the 0–20 cm soil layer were generally greater than those in the 20–40 cm layer.

### 3.7 PCA and composite score of soil factors in different herbaceous plant communities

To compare the effects of different herbaceous plant communities on soil quality and identify the main soil environmental factors, PCA was performed on the soil environmental factors in the study area. The factor loading matrix of each principal component is presented in Table 3. A total of four principal components were extracted, with the cumulative contribution rate reaching 73.994%, effectively representing the data for the sixteen soil factors. The contribution rate of principal component 1 was 26.404%, and the major soil factors included the SWC, SS, SO_4_^2–^, Cl^–^, and AK contents. The contribution rate of principal component 2 was 18.449%, and the primary soil factors were the soil CO_3_^2–^, HCO_3_^–^, TN and SOM contents. The contribution rate of principal component 3 was 15.577%, and the key soil factors were the soil Ca^2+^, Mg^2+^ and AP contents. The contribution rate of principal component 4 was 13.564%, and the primary soil factors were the BD, K^+^ content, and Na^+^ content.

**TABLE 3:** Composition matrix of different soil environmental factors after rotation.

Soil environmental factor	Principal component			
	1	2	3	4
SWC	0.726	−0.092	0.015	0.124
BD	0.026	−0.126	−0.046	0.853
SS	0.936	−0.126	0.044	0.086
pH	−0.569	0.012	0.150	−0.561
K^+^	0.454	0.218	0.431	0.524
Ca^2+^	0.002	−0.145	0.830	0.277
Na^+^	0.299	−0.192	0.447	0.653
Mg^2+^	0.493	0.114	0.598	0.422
CO_3_^2–^	0.139	0.655	−0.152	−0.036
HCO_3_^–^	−0.208	0.837	−0.231	0.025
SO_4_^2–^	0.721	−0.360	0.202	0.187
Cl^–^	0.914	−0.305	0.029	0.064
TN	−0.341	0.788	0.234	−0.095
AP	−0.100	0.064	0.824	−0.240
AK	0.600	0.360	−0.357	0.157
SOC	−0.229	0.828	0.235	−0.132
Eigenvalue	4.225	2.952	2.492	2.170
Contribution rate/%	26.404	18.449	15.577	13.564
Cumulative contribution rate/%	26.404	44.853	60.430	73.994

The weight of each principal component and the composite factor scores of each herbaceous plant community were calculated on the basis of the factor loading results of different herbaceous plant communities and the sum of the squared loadings for each principal component obtained from the PCA. The results are shown in Table 4. The analysis revealed that the ASI had the highest composite score (0.574), indicating the best soil improvement effect among the studied plant communities. In contrast, the SSA had the lowest composite score (0.373), suggesting a relatively poor soil improvement effect. The soil improvement effects of ASC and PAU were relatively high. Compared with those for SSA, the composite scores for ASI, ASC, and PAU increased by 53.89, 46.38, and 43.97%, respectively. These findings suggested that the *A. scoparia*, *A. sinensis*, and *P. australis* communities were suitable for soil improvement in the study area.

**TABLE 4:** Composite scores of the degree of membership of the soil improvement effects of different herbaceous plant communities.

Soil environmental factor	Principal component				Composite scores
	1	2	3	4	
Effect of factors	–	+	+	–	
SSA	0.435	0.115	0.551	0.402	0.373
ASI	0.834	0.197	0.500	0.664	0.574
ASC	0.774	0.392	0.437	0.438	0.546
PAU	0.784	0.270	0.504	0.461	0.537

## 4 Discussion

### 4.1 Effects of the herbaceous plant community on soil salinity

The Yellow River Delta is a primary distribution area of saline–alkaline soil in China, and soil salinization is a key factor restricting vegetation growth and ecological restoration in this region. This study revealed that the soil salinization in the Yellow River Island was relatively high, with soil pH values ranging from 7.1 to 8.1, and soil SS contents ranging from 1.0 to 12.1 g/kg. The dominant soil salts identified were sodium chloride (NaCl) and calcium sulfate (CaSO_4_). These findings were consistent with those of previous studies that reported similar soil salinity characteristics in the Yellow River Delta (Abdellaoui et al., 2023; Liu et al., 2023). However, our study further demonstrated that the soil salinity content in the herbaceous plant community was significantly lower than that of bare land, and the soil salinity content in the 0–20 cm soil layer was significantly lower than that in the 20–40 cm soil layer. This vertical stratification of soil salinity was crucial for understanding the impact of plant communities on soil quality. High concentrations of salt ions, such as Na^+^ and Cl^–^, in the soil were adsorbed by soil particles, leading to the destruction of the soil aggregate structure. This process resulted in decreased soil water retention, increased water permeability, and significant soil water loss. The loss of soil nutrients and moisture is one of the key factors contributing to soil salinization in coastal saline-alkaline lands. (Abdellaoui et al., 2023; Liu et al., 2023). Therefore, reducing the adsorption effect of salt ions and improving the water and nutrient retention capacity of the soil are effective strategies for improving saline–alkaline soils in the Yellow River Delta.

The growth of herbaceous plant communities can mitigate the adverse effects of soil salinization. In this study, the soil salinity content in herbaceous plant communities was significantly lower than that in bare land, and the salinity content in the 0–20 cm soil layer was lower than that in the 20–40 cm soil layer. Plant cover over the ground surface can suppress evaporation of surface soil moisture, thereby maintaining the concentration of salt ions in the topsoil at a relatively low level. This in turn inhibits the upward migration of salt ions from deeper soil layers (Duan et al., 2022). Simultaneously, salt ions in the soil are absorbed by the roots of halophytes due to transpiration pull and root pressure, thereby becoming incorporated into plant physiological processes. Furthermore, salt-secreting plants can release salts onto the surface of stems or leaves via salt glands (Zhu et al., 2010; Manousaki and Kalogerakis, 2011; Bentrup, 2017). In summary, plant communities play a crucial role in reducing soil salinity and improving soil quality.

In this study, in areas with relatively high soil salinity, halophytes such as *S. salsa* and *Spartina alterniflora*, can grow normally. This phenomenon is attributable to the fact that *S. salsa* and *S. alterniflora* have relatively developed salt glands and active antioxidant stress systems, enabling them to adapt to high–salt habitats (Song et al., 2022; Mittmann-Goetsch et al., 2024). Whereas in areas with low salinity, plants with relatively low salt tolerance, such as *A. scoparia*, *A. sinensis*, and *P. australis*, are better suited for growth. It is revealed that the tolerance of different herbaceous plant communities to soil salinity stress determines their survival quality in habitats with varying salinity gradients. Therefore, the selection of soil-improving plant communities for different habitat types should prioritize the survival quality of the plant communities. However, since *S. alterniflora* is an invasive species, its large–scale colonization poses a great threat to the plant community structure in its habitat (Borges et al., 2021). Therefore, in high–salinity habitats, the *S. salsa* community is suitable for desalinating and improving soil quality. In contrast, the ability of *A. scoparia*, *A. sinensis*, and *P. australis* to reduce salinity and improve soil quality was better than that of *S. salsa* in low–salinity habitats. Our study also revealed that the soil salt ion contents in plant communities, such as *A. scoparia*, *A. sinensis*, and *P. australis*, were significantly lower than those in the *S. salsa* community and bare land in low-salinity habitats. This finding also provides evidence for the preceding conclusion. Further analysis of the results revealed that plant communities with low soil salt ion concentrations in low–salinity habitats exhibited relatively high α diversities, and the β diversities among these communities were relatively high. Previous studies have shown that plant diversity and community stability are positively correlated. In low–salinity habitats, communities with high plant diversity are more effective at reducing soil salinity content and improving soil quality (Chen et al., 2025; Dítě et al., 2025). Our findings are consistent with these results, highlighting the importance of plant diversity in improving soil quality in low-salinity habitats. Therefore, enhancing plant diversity in low–salinity habitats should become a focus when improving the soil quality of coastal saline–alkaline land.

### 4.2 Improvement effect of the herbaceous plant community on soil nutrients

Soil nutrients are the source of nutrients needed for plant growth and development. The amount of soil nutrients, which indicates the level of soil fertility, determines the maturity level of plant community structure and function (Delgado-Baquerizo et al., 2017). However, salt stress suppresses the activity of the plant antioxidant stress system, inducing substantial accumulation of reactive oxygen species (ROS). This subsequently disrupts the cellular membrane structure of transport cells, impairs the function of channel proteins, thereby inhibiting the activity of enzymes associated with nutrient absorption and reducing the plant’s capacity to absorb essential nutrients (Valenzuela et al., 2022; Saleem et al., 2024; Singh et al., 2024). The ability of herbaceous plant communities to increase soil nutrient levels is a cornerstone of ecological restoration in degraded saline–alkaline landscapes. This Study have shown that areas with diverse plant communities present relatively high soil nutrient contents. This improvement is mechanistically linked to increased litter input and accelerated root–driven nutrient cycling, and this process promotes the conversion of readily available nutrients, thereby significantly improving soil fertility (Chmolowska et al., 2016; Chen et al., 2025). In the present study, we found that diverse communities—notably *A. scoparia*, *A. sinensis*, and *P. australis*, —significantly elevated the levels of SOM, TN, and AP. This is consistent with previous studies (Cao et al., 2015; Zhang et al., 2016; Zhang et al., 2023). However, one of our results presented a critical divergence from conventional wisdom: these high–diversity communities were associated with a significant decrease in soil available potassium (AK). These findings differ from the conclusions of previous studies. We contend that this phenomenon is not indicative of nutrient depletion but rather a hallmark of effective soil remediation. Research has confirmed that potassium in the saline-alkaline soils of coastal region in Yellow River Delta primarily originates from external inputs of seawater (Yu et al., 2016). Soil salinisation enhances potassium bioavailability, resulting in significantly higher AK concentrations in high-salinity habitats compared to low-salinity habitats (Xu et al., 2020). In saline soils, potassium often exists at elevated levels and can participate in complex migration and transformation processes (Mori et al., 2011). Therefore, the observed reduction in AK likely reflects its active uptake and utilization by plants for osmoregulation, a process that is vital for mitigating ion toxicity in saline environments (Yang Y. et al., 2021; Verma et al., 2025). This result suggests that in the context of saline soil reclamation, a decrease in AK can be a positive indicator of restored plant–soil interactions and improved potassium cycling efficiency, rather than a nutrient deficiency. In summary, plant community selection on Yellow River Island should not solely target nutrient addition but should also consider the regulation of pre–existing, potentially excessive, ion pools, and reasonable plant allocation, which can be based on soil salinity reduction and soil improvement effects. This nuanced understanding allows us to move beyond generic recommendations.

For instance, while our PCA and factor composite score analysis revealed that the *A. sinensis* community was the most effective for overall soil quality improvement on Yellow River Island, practical applications demand a spatially explicit strategy. Furthermore, the *A. scoparia* and *P. australis* communities effectively improved soil quality. However, the ability of these plant communities to tolerate high–salinity habitats is limited. Plant communities such as *S. salsa* can increase the root uptake of salt ions and secretion through salt glands by releasing large amounts of ROS because of increased antioxidant enzyme activity; therefore, the community can better adapt to saline habitats (Guan et al., 2017; Song et al., 2022). Therefore, in high–salinity habitats, priority should be given to using more salt–tolerant halophytic plant communities, such as *S. salsa*, *P. australis*, and *S. alterniflorus*. A study on soil quality improvement in the Yellow River Delta Nature Reserve revealed that the halophyte communities of glassworts and alfalfa demonstrated favorable soil improvement effects (Xia et al., 2019). A previous study also revealed that the *P. australis* community achieved the best soil improvement effect in Binzhou Port, and recommended that community allocation should consider a combination of halophytes, such as *S. salsa* and *Oxybasis glauca* (Yang H. et al., 2021). In summary, plant community allocation for soil improvement must account for spatial heterogeneity in soil salinity. Binzhou Port and the Yellow River Delta Nature Reserve, which are near coastlines with inherently high soil salinity, necessitate a primary focus on halophyte–dominated communities for ecosystem engineering and initial soil stabilization. In contrast, a zonal allocation strategy is essential for Yellow River Island, whereby the selection of plant functional types is tailored according to the spatial heterogeneity of soil salinity.

## 5 Conclusion

The improvement effects of different herbaceous plant communities on soil quality in the Shandong Yellow River Island National Wetland Park vary significantly. The communities of *A. scoparia*, *A. sinensis*, and *P. austral* is exhibit relatively high levels of α– and β diversity, along with greater plant species richness and community similarity. These herbaceous plant communities effectively increase soil quality within the 0–20 cm soil layer. In terms of salinity reduction, communities with higher diversity levels demonstrate superior desalination effects compared with others, with the *A. scoparia* community showing the strongest performance. Herbaceous plant communities reduce soil salinization by regulating the enrichment of salt ions, such as Na^+^, Cl^–^, and SO_4_^2–^. With respect to soil nutrient improvement, the *A. sinensis* and *A. scoparia* communities have the greatest effects on increasing soil TN and AP, respectively. Furthermore, both the *A. sinensis* and *P. australis* communities contribute significantly to increasing SOM. The results of PCA and composite factor score analysis confirmed that the *A. scoparia*, *A. sinensis*, and *P. australis* communities are effective at improving soil quality on Yellow River Island. Consequently, we critically report that the optimal restoration strategy involves a targeted community approach on Yellow River Island: deploying a mixed community of *A. scoparia*, *A. sinensis* and *P. australis* near waterways and inland saline–alkaline regions, and a combination of *S. salsa* and *P. australis* communities in muddy coastal zones. This targeted approach ensures that restoration efforts are both ecologically sound and cost effective.

## Data Availability

The original contributions presented in the study are included in the article/supplementary material, further inquiries can be directed to the corresponding authors.
